# Environmental resistome of culturable gram-negative bacilli from the Yaque del Norte River: baseline genomic evidence for one health surveillance

**DOI:** 10.3389/fmicb.2026.1780969

**Published:** 2026-04-21

**Authors:** María F. Chevalier-Alba, Dionis I. Hoepelman, Larianna Pineda-Cabrera, Lázaro M. Acosta-Rivera, Seudimar Blanco, Roberto Bonelly, Holly Rosario, María Gonzalez, Dairelys Guerrero, Irene Ortiz, Argeny Ovando, Claudia Reyes, Luis Enrique Rodríguez de Francisco, Rommel Ramos, Luis O. Maroto-Martín, Edian F. Franco

**Affiliations:** 1 Instituto Tecnologico de Santo Domingo (INTEC), Santo Domingo, Dominican Republic; 2 Research and Innovation Department, Instituto de Innovacion en Biotecnologia e Industria (IIBI), Santo Domingo, Dominican Republic; 3 Instituto Superior de Formación Docente Salomé Ureña (ISFODOSU), Santo Domingo, Dominican Republic; 4 Universidad ISA (UNISA), Santiago de los Caballeros, Dominican Republic; 5 Centro de Investigación, Facultad de Ciencias de la Salud, Universidad Tecnológica de Santiago (UTESA), Santo Domingo, Dominican Republic; 6 Laboratory of Bioinformatics and Genomics of Microorganisms, Institute of Biological Sciences, Federal University of Pará, Belém, Pará, Brazil; 7 Escuela de Informatica, Facultad de Ciencias, Universidad Autonoma de Santo Domingo (UASD), Santo Domingo, Dominican Republic

**Keywords:** Cibao, Dominican Republic, *Enterobacteriaceae*, multidrug resistance, Yaque del Norte River

## Abstract

Antimicrobial resistance (AMR) represents a significant global public health threat. Aquatic ecosystems are increasingly recognized as critical reservoirs of resistance determinants within the One Health framework. Freshwater systems, in particular, facilitate the persistence and dissemination of antimicrobial resistance genes (ARGs) at the human–animal–environment interface. This study characterized the environmental resistome of culturable Gram-negative bacilli isolated from the Yaque del Norte River, the largest river in the Dominican Republic. Water samples were collected along the river basin to assess physicochemical parameters and recover bacterial isolates. Isolates were identified using MALDI-TOF MS and JspeciesWS. Whole-genome sequencing and bioinformatic analyses with CARD and the Galaxy staramr toolkit were performed to identify ARGs and plasmid replicons. Pathogenic potential was evaluated using PathogenFinder v1.1. Six bacterial strains were recovered: *Pseudomonas aeruginosa*, *Klebsiella pneumoniae*, *Klebsiella quasipneumoniae*, *Enterobacter mori*, *Acinetobacter pittii*, and *Acinetobacter schindleri*. A total of 112 ARGs were identified, with P. aeruginosa harboring the most extensive resistome, comprising 51 genes. ARG identification was based on sequence identity ≥ 90% and coverage ≥ 80% according to CARD and ResFinder criteria. Multidrug resistance–associated determinants were predominant, particularly efflux pump systems and transcriptional regulators. Additional resistance mechanisms included genes conferring resistance to fluoroquinolones, β-lactams, bacitracin, fosfomycin, and aminoglycosides. Plasmid replicons were detected exclusively in *Klebsiella* spp. Both species carried IncFIB(K), and one isolate harbored IncY, a replicon rarely reported outside *Escherichia coli* and *Salmonella* serovar Typhi, suggesting potential interspecies dissemination. Phenotypic resistance was observed only in a single *K. pneumoniae* isolate. Nevertheless, the presence of diverse ARGs in this environmentally impacted river underscores the potential for future emergence and spread of AMR. These findings provide baseline genomic evidence to support environmental AMR surveillance and highlight the need to strengthen antibiotic stewardship and One Health–oriented monitoring strategies in the Dominican Republic.

## 1 Introduction

Gram-negative bacilli, including *Enterobacteriaceae*, span over 50 genera and about 200 species (Jenkins et al., 2017). These bacteria thrive globally in environments such as soil, plants, and water, growing rapidly in both aerobic and anaerobic conditions (Jenkins et al., 2017; Riedel et al., 2019a,b). Several Gram-negative bacteria are pathogenic or opportunistic, impacting human and animal health (Jenkins et al., 2017; Zhang and Wang, 2023). Notably, under a One Health framework, freshwater ecosystems serve as critical interfaces, linking environmental reservoirs to human and animal exposure pathways. This interconnectedness makes them priorities for environmental antimicrobial resistance (AMR) surveillance (World Health Organization [WHO], 2014, 2024b). Scientific and healthcare communities are increasingly concerned about antimicrobial-resistant Gram-negative strains (World Health Organization [WHO], 2014, 2024b), which often cause gastrointestinal problems such as dehydration and diarrhea in humans and animals (Jenkins et al., 2017). Notable pathogenic and opportunistic strains include *Salmonella enterica* serovar Typhi, *Shigella dysenteriae*, enterotoxigenic *Escherichia coli* O157:H7, *Yersinia pestis*, *Klebsiella pneumoniae*, and *Enterobacter cloacae* (Jenkins et al., 2017; Yin et al., 2025).

Nowadays, these bacteria are more infectious because of new mechanisms that confer antibiotic resistance (Yin et al., 2025). The most commonly used mechanisms are enzymatic inhibition, modifications to penicillin-binding proteins (PBPs), porin mutations, efflux pumps, and target changes (Kon and Rai, 2016). For example, enzymatic inhibition involves enzymes such as β-lactamases, which hydrolyze the β-lactam ring of penicillins (MacGowan and Macnaughton, 2017). As *Enterobacteriaceae* produce more β-lactamases, their infections become harder to treat, leading to growing resistance to penicillins and cephalosporins (Jenkins et al., 2017; MacGowan and Macnaughton, 2017). In addition, carbapenemases, another type of enzyme, are spreading widely and can inhibit all β-lactam agents except aztreonam (Kon and Rai, 2016; MacGowan and Macnaughton, 2017).

All resistance mechanisms are encoded by gene complexes that cluster into the antibiotic and metal resistome, which includes antibiotic resistance genes (ARGs), their precursors, metal resistance genes (MRGs), and plasmids (Chernova et al., 2021; Kim and Cha, 2021). Resistomes can be either native or acquired, as genetic material may be transmitted vertically from parent to offspring or horizontally between unrelated cells (Mourão et al., 2025). Horizontal transfer takes place via transformation, transduction, or conjugation, with plasmid-mediated conjugation being most effective in aquatic environments (Bingle and Thomas, 2001; Bennett, 2008; Chernova et al., 2021).

These environments are ideal hotspots for gene transfer. Bodies of water are already prone to pollutants, which commensal bacteria use to develop (Abe et al., 2020; Mourão et al., 2025). Such pollutants include unmanaged antibiotics. After excretion, antibiotics may remain unchanged or form metabolites that are equally harmful. These compounds enter wastewater systems and contaminate the water they contact (Chouchani et al., 2013). Aquatic ecosystems contain bacteria that form microcolonies, called biofilms. Biofilms foster the proliferation and exchange of antibiotic-resistant genes. This propagates resistomes through horizontal gene transfer among microcolony members (Abe et al., 2020; Mourão et al., 2025).

Yaque del Norte is the largest and most important waterway in the Dominican Republic. This 296 km river runs through the northern region (El Cibao), and supplies water for agriculture, energy, and drinking (Acosta Guzmán, 2015). However, anthropogenic practices have weakened waste management over the last decade (United Nations, 2025). With poor sewage and wastewater treatment, waste leaks into rivers and oceans. And in turn, this could favor antibiotic resistance, causing AMR to spread to non-resistant strains (Singh et al., 2022).

In this study, we aim to describe the environmental resistome of culturable Gram-negative bacilli in the Yaque del Norte River. This study aimed to characterize potential environmental reservoirs of antibiotic resistance genes and plasmids in the Yaque del Norte River to provide baseline genomic evidence for One Health surveillance. This objective was pursued by providing initial genomic information on these bacterial taxa and their resistomes; how they might affect the surrounding population; and associating our findings with the national reports addressing pathogenic and resistant bacteria emitted by the Dominican Republic.

## 2 Experimental procedure

### 2.1 Sampling locations and methods

**FIGURE 1:**
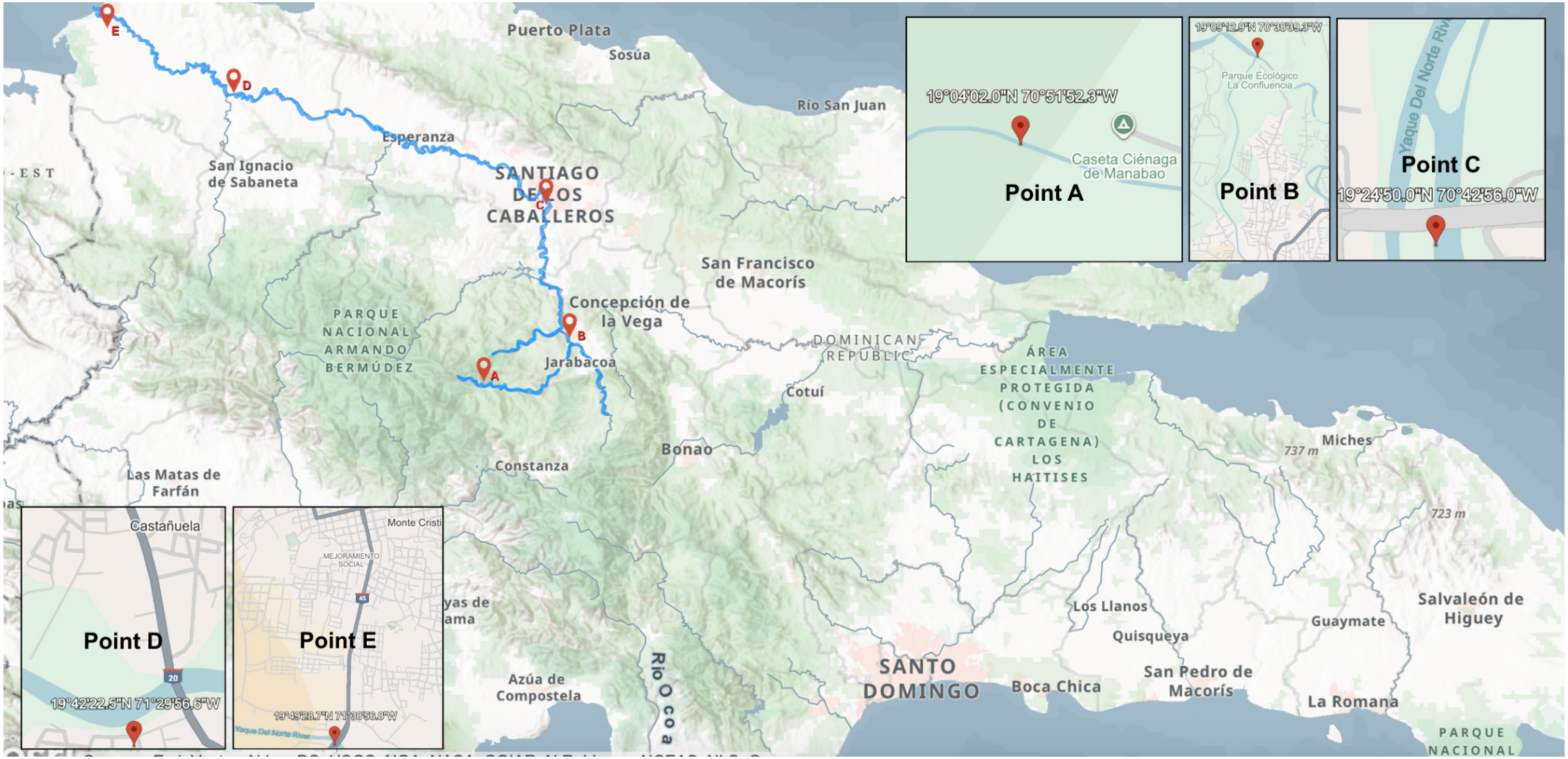
Map of the Dominican Republic, highlighting the Yaque del Norte River (in blue), with the sampling location points: A (Manabao), B (Jarabacoa), C (Otra Banda), D (Castañuela) and E (Monte Cristi). Modified from Esri (2025) and Google (2025).

Water samples from the Yaque del Norte River were collected as biological triplicates on February 2022 at five locations along the basin. A Van Dorn bottle (1.5 L; Hydro-Bios) was used for collection. The locations were: Manabao, La Vega (Point A; 19°04′02.2″N, 70°51′52.3″W); Confluence, Jarabacoa (Point B; 19°09′11.2″N, 70°38′35.2″W); Otra Banda, Santiago (Point C; 19°27.266″N, 70°42.981″W); Castañuela, Monte Cristi (Point D; 19°42′22.4964″N, 71°29′56.562″W); and Monte Cristi (Point E; 19°49′28.7″N, 71°38′56.8″W), these are showcased in Figure 1. The locations were selected based on the river’s flow, from the source (Point A) to the mouth (Point E), noting the selection points were chosen based on the closest possible access point allowed by the *Ministerio de Medio Ambiente y Recursos Naturales*. The observable physical characteristics of the river sampling points are described below in Table 1. All samples were kept at 4°C during transport. They were delivered to the Instituto de Innovación en Biotecnología e Industria (IIBI) in Santo Domingo for analysis.

**TABLE 1:** Characteristics of the sampling sites.

Basin	Altitude and location	Latitude and longitude	Characteristics of the zone	Level of impact
Upper	Manabao (1,135 m a.s.l.)	N 19° 35’23.4312” W 71° 03’34.6752”	This sampling point is the closest accessible site to the river headwaters, in the Armando Bermúdez National Park. The water is clear and its use is restricted within the park, but outside the protected area, the water is used for the agriculture and domestic purposes of the population (approx. 5,000 inhabitants).	Low
Middle	Confluence (Yaque del Norte and Jimenoa) (481 m a.s.l.)	N 19°09.216’ W 070°38.655”	This point was used to evaluate the microbial load of the Yaque del Norte river after encountering the Jimenoa river. This water is used for domestic purposes, for residential and recreative areas are located near the site (approx. 40, 556 inhabitants).	Medium
Middle	Otra Banda (Puente Japur Dumit) (181 m a.s.l.)	N 19°27.266’ W 070°42.981”	This site is located near the Ulises F. Espaillat and Monsieur Boagaet canals, which supply irrigation to various communities such as La Canela, Hato del Yaque, Navarrete and Villa González, among others. The water had was turbid and organic and solid waste were observed, as well as abundant algae growth. Its densely populated surroundings include communities and farms that use the water for fishing and domestic purposes, and discharge their wastewater into the river, without prior treatment (approx. 1,343,423 inhabitants).	High
Middle	Mao	N 19° 35’23.4312” W 71° 03’34.6752”	Contamination with solid and organic waste could be seen in the waters, as well as the multiple residences and cultivated areas in which the river’s water is used for irrigation (approx. 76,863 inhabitants).	Medium
Lower	Castañuela	N 19° 42’22.4964” W 71° 29‘56.562”	The water was characterized by being turbid and being surrounded by multiple residences and farms (approx. 14,900 inhabitants).	Medium
Lower	Monte Cristi	N 19° 49’45.5268” W 71° 38’52.3212”	The water from this site was turbid with no visible solid waste. Although farms or residences were not observed near the area, the water is used for fishing and the irrigation of nearby crops (approx. 150,000 inhabitants).	Medium

### 2.2 Determination of the physical-chemical parameters

Physical-chemical parameters were measured *in situ* at each sampling point using a rugged, waterproof multiparameter meter (HI98194; IP67). The instrument was equipped with a digital probe and sensors to record temperature, pH, oxidation-reduction potential (ORP), electrical conductivity (EC), and total dissolved solids (TDS). Measurements were recorded using the instrument’s data-logging function, and calibration and electrode diagnostic checks (CAL Check™) were performed according to the manufacturer’s recommendations prior to field deployment. Biochemical oxygen demand (BOD) was determined using a respirometer (BOD Track II). Additional parameters, when applicable, were obtained through further analyses conducted by the Instituto Nacional de Recursos Hidráulicos (INDRHI).

All physical-chemical analyses were performed in triplicate. Accepted ranges followed the Normas Ambientales de Agua Superficiales y Costeras and the Dirección de Asuntos Ambientales y Cambio Climático at the national level. International guidelines as posted by the WHO were also used (Ministerio de Medio Ambiente y Recursos Naturales, 2012; Gobierno de la República Dominicana, 2021; Latif et al., 2024; World Health Organization [WHO], 2024a).

### 2.3 Isolation and bacteria culture

River water samples were processed directly to obtain well-isolated colonies and enable meaningful chromogenic differentiation. Serial dilutions were prepared up to 10^–5^. For each dilution, 1 mL of the sample was inoculated into CHROMagar™ Orientation (prepared according to the manufacturer’s instructions) using the pour-plate method: the inoculum was mixed with molten medium cooled to approximately 45–50°C, then poured into 96-mm Petri dishes (15–20 mL medium per plate). Plates were incubated aerobically at 35–36°C for 24 h. CHROMagar™ Orientation is a chromogenic differential medium designed for the qualitative detection and presumptive differentiation of uropathogens based on colony color patterns generated by enzymatic activities acting on chromogenic substrates; in this study, it was used as an initial chromogenic screening medium to facilitate colony selection from an environmental water matrix, and definitive identification was subsequently confirmed by MALDI-TOF MS and whole-genome sequencing.

Identifiable colonies were selected based on differentiated criteria, including color, opacity, size, edge irregularity, and elevation, and were subcultured onto separate Petri dishes. Furthermore, isolates belonging to the *Enterobacteriaceae* family were preferentially selected for the study, and from the chromogenically identified enterobacteria, isolates that presented known risks and are part of the WHO’s Bacterial Priority Pathogen List were chosen for further sequencing. Pure cultures were obtained by streaking using sterile bacteriological loops. Uninoculated plates were incubated in parallel as negative controls to monitor for environmental contamination.

### 2.4 Bacteria identification

The isolates were identified using MALDI-TOF and Vitek from Laboratorio Clínico Referencia in Santo Domingo, Dominican Republic, and further characterized with JSpeciesWS Online Service (Richter et al., 2016). The species were classified based on a z-score of 0.999; however, for those that did not meet this criterion, the Type (Strain) Genome Server (TYGS) was used to compare them with other members of the genus (Meier-Kolthoff and Göker, 2019). It is also worth noting that the analysis conducted by the Laboratorio Clínico Referencia was complemented by a minimum inhibitory concentration (MIC) assay for certain antibiotics. For which the tested formulations of antibiotics were amikacin, ampicillin–sulbactam, cefepime, ceftriaxone, ciprofloxacin, ertapenem, gentamicin, meropenem, piperacillin–sulbactam, levofloxacin, tazobactam, trimethoprim–sulfamethoxazole (TMP–SMX), and tigecycline. The protocol followed by the laboratory was performed using the BD Phoenix M50 automated system (Becton Dickinson, United States) with the ACT-NMIC406 panel. The MIC tests were carried out by broth microdilution methodology. Results were interpreted according to the CLSI guidelines followed by the laboratory (CLSIM100-2021). Quality control was performed using *Escherichia coli* ATCC 25922, *Escherichia coli* ATCC 35218, *Klebsiella quasipneumoniae* ATCC 700603, and *Pseudomonas aeruginosa* ATCC 27853. The quality control standard was selected accordingly for each bacterium, in the case of *Acinetobacter* sp. and *Enterobacter mori, E. coli* was the quality control.

### 2.5 DNA extraction

Using the commercial Qiagen DNeasy Blood and Tissue kit, the genomic DNA was extracted from the 6 identified isolates using the MALDI-TOF equipment. The instructions were followed as stated by the supplier, with modifications: initial lysis at 56°C for 1 h and final elution with 100 μL of AE buffer (Qiagen).

DNA concentration was determined using the Qubit 4.0 fluorometer (Thermo Fisher Scientific) with the dsDNA HS Assay Kit, and NanoDrop 2000 was used to assess quality, establishing a 1.8–2.0 A260/280 ratio as the acceptable range. Integrity was verified by 1% agarose gel electrophoresis stained with GelRed, conducted at 90 V for 45 min in a GELATO chamber.

### 2.6 DNA sequencing and genome assembly

The DNA was sent for sequencing at America Novogene Bioinformatics Technology Co., Ltd. in San Diego, California, United States. The libraries were built using approximately 350 bp inserts and sequenced with the Illumina NovaSeq 6,000, generating paired-end reads of 150 bp (2 × 150 cycles). The protocol followed by Novogene included genomic DNA fragmentation by sonication, followed by end-polishing, A-tailing, and ligationwith full length adapters of Illumina sequencing. Then, further PCR amplification with P5 and indexed P7 oligos was carried out, with a final purification using the AMPure XP system.

The raw archives in FASTQ format were submitted to FastQC v0.11.9 and NGS QC Toolkit v2.3.3 for quality control (Andrews, 2025; Patel and Jain, 2015), and the adapters and low-quality reads (Q < 30, longitude < 50 bp) were eliminated with Trimmomatic v0.39 (Bolger et al., 2014; Nitnaware et al., 2021). The K-mer distribution was analyzed with KmerStream, and genomes were assembled with the default parameters of the Shovill (Version 1.4.2) Galaxy toolkit, which includes SPAdes software coupled with trimmed reads to assess data quality (Seemann, 2025). Finally, the Quality Assessment Tool for Genome Assemblies (QUAST) v5.3.0 from Galaxy was employed using default settings.

### 2.7 Genome annotation

The assembled genomes were annotated with the Rapid Annotation using Subsystems Technology (RAST) tool and evaluated for assemblage metrics (N50, L50, genome size, number of contigs) using QUAST v5.0 (Aziz et al., 2008; Gurevich et al., 2013).

### 2.8 Resistome and pathogenicity potential identification

The assembled genomes were studied with the Comprehensive Antibiotic Resistance Database (CARD) for the determination of resistance genes, and the Global Pathogens Analysis Platform to identify plasmids using PlasmidFinder v2.1. (enterobacteriales database, 95% identity, 80% coverage); and other resistance genes using ResFinder v4.7.2. (90% identity, 80% min. length, show unknown mutations, and apply model strain if available) (Camacho et al., 2009; Carattoli et al., 2014; Bortolaia et al., 2020). For the construction of the ARG co-occurrence network, the identified ARG–species associations were organized in a spreadsheet using Microsoft Excel. A binary presence–absence matrix was generated, where each isolate was associated with the ARGs detected in its genome. The file was exported in CSV format and imported into Cytoscape Web v1.0.6 for network visualization. Pathogenicity potential was evaluated with PathogenFinder2 v6.0 (all extra phenotyping analysis were enabled) (Cosentino et al., 2013). The CARD outputs were considered positive when the genes had an identity percentage ≥ 90% and a coverage ≥ 80%.

## 3 Results

### 3.1 Physical-chemical properties of the river

The physical-chemical analysis showed that temperature, pH, and ORP were within the accepted ranges set by Dominican standards for surface water. However, EC, TDS, and BOD exceeded recommended thresholds at several sites, both by national and international standards. The results of the analyzed parameters are summarized in Table 2.

**TABLE 2:** Physical-chemical parameter values of the Yaque del Norte River for each of the sampled points.

Parameters	Point A	Point B	Point C	Point D	Point E
Temperature (*^o^*C)	17.9	24.6	29.3	31.6	29.6
pH	8.5	8.2	7.7	7.8	7.95
ORP (mV)	167.33	184.27	184.57	221.43	218.63
BOD (mg/L)	4.8	6	6.3	12.9	8.7
EC (μS/cm)	40.5	131	203	996.33	1188.33
TDS (mg/L)	35.7	63.1	114.2	470	553

These results show a progressive increase in the analyzed parameters downstream. Point A corresponds to the upper basin, whereas Points B–E represent intermediate to lower sections of the river. These points reflect increasing human influence that correlates to the description of the surrounding areas mentioned before.

### 3.2 Bacteria isolates

Six bacterial strains were isolated from the Yaque del Norte River and selected for identification through preliminary MALDI-TOF MS analysis, followed by whole-genome–based comparison using the JSpeciesWS platform (Table 3).

**TABLE 3:** Bacteria isolated from the Yaque del Norte River collection points, and identification classification through MALDI-TOF and JspeciesWS.

Codes	Location point	MALDI-TOF	JspeciesWS	Z-Score JspeciesWS
*AP*	A	*Acinetobacter pittii*	*Acinetobacter pittii*	*0.99942*
*ASH*	D	*Acinetobacter baumannii*	*Acinetobacter schindleri HOEPLIER*	*0.99776*
*EM*	E	*Enterobacter cloacae*	*Enterobacter mori LMG 25706*	*0.99933*
*KQP*	D	*Klebsiella pneumonae*	*Klebsiella quasipneumonae*	*0.99978*
*KP*	B	*Klebsiella pneumonae*	*Klebsiella pneumonae*	*0.99991*
*PA*	D	*Pseudomonas aeruginosa*	*Pseudomonas aeruginosa*	*0.99995*

Genome-based analyses refined several initial identifications, highlighting the importance of WGS for accurate taxonomic resolution in environmental isolates. The isolate initially identified by MALDI-TOF as *Acinetobacter baumannii* was reassigned by TYGS analysis to *Acinetobacter schindler*i, confirming its genetic distinctness from *A. baumannii*. The TYGS also reported the isolate as a possible new strain from the *A. schindleri* species, hence the strain code HOEPLIER. No culturable Gram-negative isolate meeting selection criteria was recovered from Point C (Santiago).

The minimum inhibitory concentration (MIC) assays showed that most isolates were phenotypically susceptible to the tested antibiotics, except for one *Klebsiella pneumoniae* isolate, which exhibited resistance to ciprofloxacin and trimethoprim-sulfamethoxazole.

### 3.3 Antibiotic resistant genes

**FIGURE 2:**
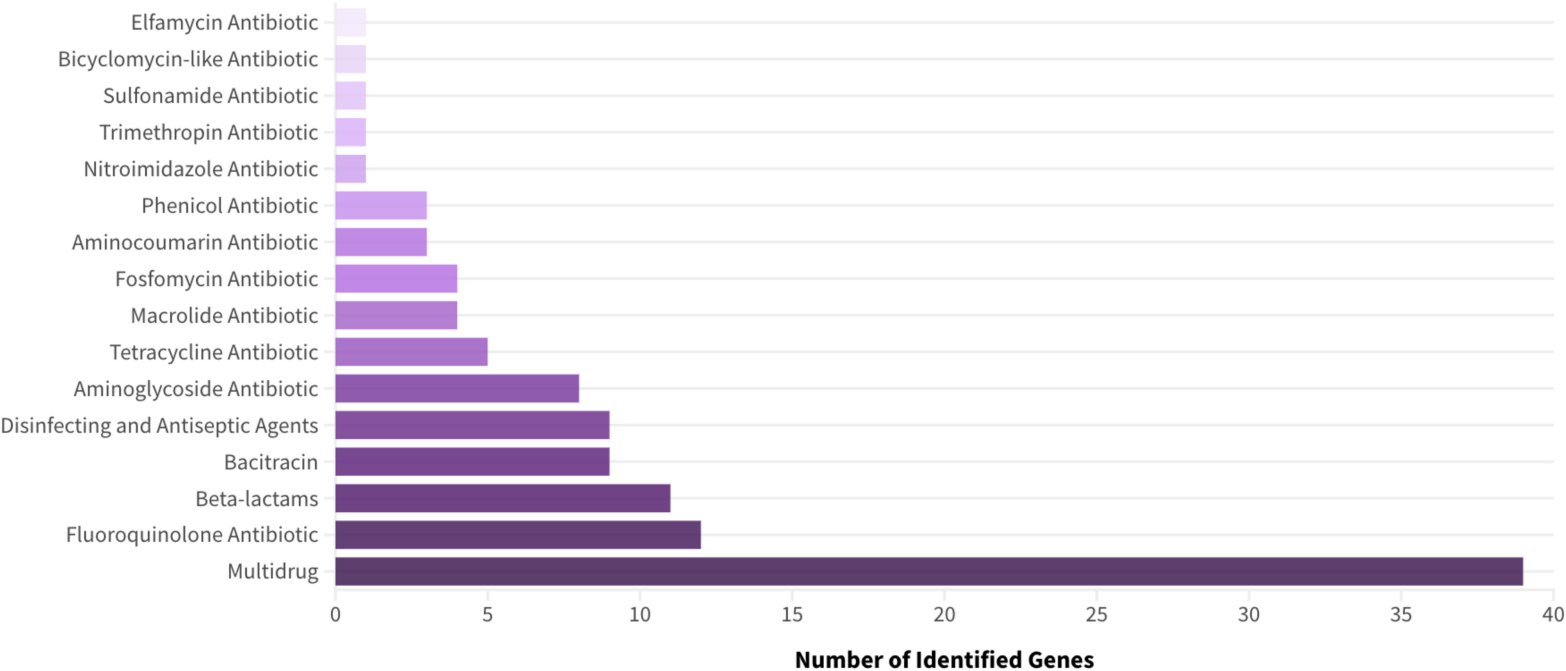
Number of resistance genes identified using CARD and ResFinder, classified by antibiotic class. Multidrug resistance genes represent the most abundant category.

The isolates had diverse environmental resistomes. *Acinetobacter schindleri* (HOEPLIER strain) carried only one detected ARG, while other taxa showed more complex profiles. Across all six genomes, 112 antibiotic resistance genes (ARGs) were identified (Table 4). These are grouped by drug class in Figure 2

**TABLE 4:** Identified resistance and resistance-associated genes in each strain.

Drug class	*A. pittii*	*K. pneumoniae*	*P. aeruginosa*	*Acinetobacter* sp.	*K. quasipneumoniae*	*E. mori*
Multidrug	NI	*H-NS, marA*, Klebsiella pneumoniae *KpnF*, Klebsiella pneumoniae *KpnE*, Klebsiella pneumoniae *KpnH*, Klebsiella pneumoniae *KpnG*, *oqxA, oqxB, LptD, CRP*	*rsmA, OprM, opmE, mexQ, mexP, YajC, MexV, MexW, OprJ, MexD, MexC*, Pseudomonas aeruginosa *soxR, mexY, MuxA, MuxB, MuxC, OpmB, OprN, MexF, MexE, ParS, ParR*, Pseudomonas aeruginosa *CpxR, Type A NfxB, MexZ, MexT, MexS*	NI	*H-NS, LptD, oqxA*, Klebsiella pneumoniae *KpnG*, Klebsiella pneumoniae *KpnH*, *CRP, marA*, Klebsiella pneumoniae *KpnE*, Klebsiella pneumoniae *KpnF*, Klebsiella pneumoniae *acrR* with mutation conferring multidrug antibiotic resistance	*oqxA, marA, H-NS, CRP*, Escherichia coli *AcrAB-TolC* with *MarR* mutations conferring resistance to ciprofloxacin and tetracycline
Bacitracin	*LpsB*,	*OmpA, ArnT, eptB*	*arnA, basS, cprR, cprS, basR*	NI	*OmpA, eptB, ArnT*	NI
Fluoroquinolone antibiotic	Acinetobacter baumannii *AbaQ*, *adeL, adeF*	*emrR, QnrB10, QnrB19*	*PmpM, OpmD, MexI, MexH*	NI	*emrR*	*emrB, emrR, qnrE1*
Macrolide antibiotic	*abeS*, Acinetobacter baumannii *AmvA*	NI	*MexK, MexJ*	NI	NI	NI
Aminocoumarin antibiotic	*abeS*	*baeR*	NI	NI	*baeR*	*baeR, baeS*
Tetracycline antibiotic	*adeL, adeF, tet(39)*	NI	*MexK, MexJ*	NI	NI	NI
β-lactams	*ADC-18, OXA-500*	*MdtQ, SHV-61*, Klebsiella pneumoniae *OmpK37*	*OXA-486, PDC-565, blaPAO*	*OXA-646*	*OKP-A-12*	*ACT-156*
Aminoglycoside antibiotic	*ANT(3”)-IIa*	*aadA2, baeR*	Pseudomonas aeruginosa *emrE, PmpM, APH(3’)-IIb*	NI	*APH(6)-Id, aph(3”)-Ib, baeR*	*baeR, baeS*
Disinfecting agents and antiseptic	Acinetobacter baumannii *AmvA*	*qacE*Δ*1*	*OpmH, PmpM, TriB, TriC, OpmD, MexI, MexH*	NI	NI	NI
Nitroimidazole antibiotic	NI	*msbA*	NI	NI	*msbA*	*msbA*
Phenicol antibiotic	NI	NI	*mexM, mexN*, Pseudomonas aeruginosa *catB7*	NI	NI	NI
Trimethropin antibiotic	NI	*dfrA12*	NI	NI	NI	NI
Fosfomycin antibiotic	NI	*FosA6*, Escherichia coli *UhpT* with mutation conferring resistance to fosfomycin	*FosA*	NI	*FosA6*, Escherichia coli *UhpT* with mutation conferring resistance to fosfomycin	*FosA2*
Sulfonamide antibiotic	NI	*sul1*	NI	NI	NI	NI
Bicyclomycin-like antibiotic	NI	NI	*bcr-1*	NI	NI	NI
Elfamycin antibiotic	NI	NI	NI	NI	NI	Escherichia coli *EF-Tu* mutants conferring resistance to Pulvomycin

NI, None identified.

*Acinetobacter pittii* presented a small resistome (10 ARGs) without MDR-associated genes. *Enterobacter mori* harbored 14 ARGs, including those linked to MDR and resistance to fluoroquinolones, β-lactams, and elfamycins. *Klebsiella pneumoniae* and *Klebsiella quasipneumoniae* had larger resistomes, with 27 and 21 ARGs, respectively. The *K. pneumoniae* isolate showed a balanced distribution of MDR, fluoroquinolone, bacitracin, and β-lactam resistance genes. In contrast, *K. quasipneumoniae* predominantly carried MDR-associated genes, followed by resistance genes for bacitracin, aminoglycosides, and fosfomycin.

*Pseudomonas aeruginosa* had the largest resistome, with 51 identified ARGs. Most were associated with MDR, but some conferred resistance to individual antibiotic classes. Only these four taxa showed MDR-associated determinants, indicating more complex resistome profiles in taxa frequently associated with human and animal environments.

**FIGURE 3:**
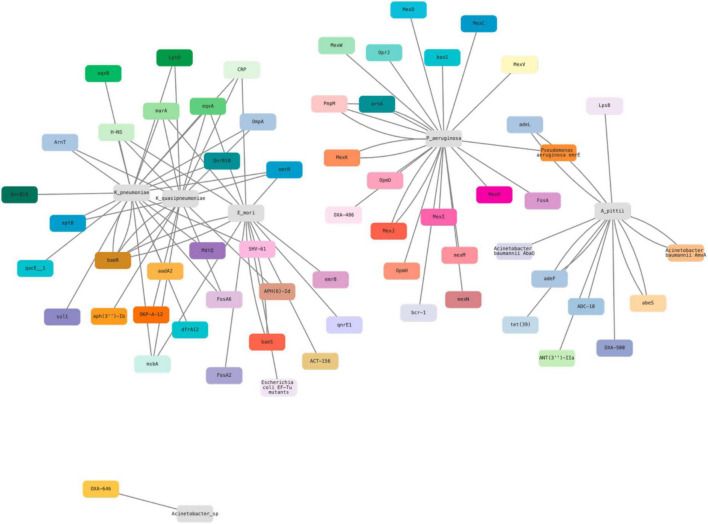
ARG co-occurrence network generated using Cytoscape Web v1.0.6 based on ARG presence across isolates.

All the identified ARGs were combined and compared against each strain, and represented in an ARG co-occurrence network, as shown in Figure 3.

### 3.4 Plasmid and pathogenic strain identification

PlasmidFinder analysis revealed plasmid replicons in only *Klebsiella* isolates Table 5. Specifically, both *K. pneumoniae* and *K. quasipneumoniae* carried *IncFIB(K)* replicons, while *K. quasipneumoniae* also had *CoIE10*, *IncR*, and *IncY* plasmids. Together, these findings suggest a higher potential for gene transfer in this taxon.

**TABLE 5:** Identified plasmids in each sample.

Strain	Plasmid	Identity	Accession number
*KQP*	*ColE10*	99.44	X01654
	*IncR*	99.6	DQ449578
	*IncY*	99.48	K02380
	*IncFIB(K)*	98.75	JN233704
*KP*	*IncFIB(K)*	99.46	JN233704

PathogenFinder analysis predicted that all six isolates have a moderate to high likelihood of pathogenicity. This indicates the presence of genomic features linked to human pathogenicity, shown in Table 6. These predictions do not imply clinical pathogenicity but highlight the relevance of these environmental bacteria as potential reservoirs of virulence- and resistance-associated traits.

**TABLE 6:** Bacteria pathogenic Capacity according to PathogenFinder2.

Strain	Bacteria pathogenic capacity	Closest reference pathogenic NEIGHBOR	Minkowski distance to reference bacteria	Pathogen
*AP*	0.9753	*Acinetobacter* sp. TUM15378 (GCF_009001635.1)	0.06868358701467514	✓
*ASH*	0.9661	*Acinetobacter baumannii* (GCF_023001605.1)	0.023124152794480324	✓
*EM*	0.9573	*Leclercia adecarboxylata* (GCF_004295325.1)	0.05412759259343147	✓
*KQP*	0.9772	*Klebsiella pneumoniae* (GCF_018137855.1)	0.026814628392457962	✓
*KP*	0.9773	*Klebsiella pneumoniae* (GCF_002851575.1)	0.05370965227484703	✓
*PA*	0.9871	*Pseudomonas aeruginosa* (GCF_003977895.1)	0.039876095950603485	✓

## 4 Discussion

### 4.1 Antibiotic resistance genes

Along the four locations sampled in the Yaque del Norte River, resistant strains of *Klebsiella pneumoniae* and *Enterobacter* sp. were detected, suggesting the presence of a reservoir of bacteria with pathogenic potential. The *Enterobacter* sp. strain, even though identified as *E. cloacae* by the MALDI-TOF equipment, couldn’t be identified at a species level by the JspeciesWS software, meaning that the MALDI-TOF identification most likely resulted in an approximation to the closest strain available in their library (Lasch et al., 2025).

All strains included resistance genes in their genomes, resulting in broad resistomes that encompass resistance determinants associated with multiple antibiotic classes. As for antibiotic resistance, the four strains harbored a large number of genes associated with multidrug, fluoroquinolone, β-lactam, aminoglycoside, and fosfomycin resistance.

Efflux-based resistance mechanisms were widely represented among the isolates, with identified genes belonging mainly to the Resistance–Nodulation–Division (RND), Small Multidrug Resistance (SMR), Major Facilitator Superfamily (MFS), and Porin/Outer Membrane Factor (OMF) families (Elshobary et al., 2025; Vivekanandan et al., 2025). These systems contribute to reduced intracellular accumulation of antimicrobial compounds and are closely linked to bacterial metabolism, pathogenesis, and resistance phenotypes. In Gram-negative bacteria, the outer membrane acts as a critical barrier that regulates compound transport, and alterations in porin expression encoded by the *opr* and *omp* genes, detected in this study, limit antibiotic penetration through the semipermeable membrane (Muderris et al., 2018). Such permeability changes act synergistically with efflux systems, including the Mex pumps identified here, and are frequently associated with β-lactam resistance profiles (Galgano et al., 2025).

*oqxA*/*oqxB* (OqxAB) was identified as a member of the RND family, a major efflux system group in Gram-negative bacteria that contributes not only to antimicrobial resistance but also to the extrusion of toxins and metabolic by-products, consistent with the idea that these pumps are known to participate in detoxification processes and have been implicated in antibiotic resistance under selective pressure (Vivekanandan et al., 2025). OqxAB, originally described on the pOLA52 plasmid, is highly prevalent in Enterobacteriaceae and has been associated with resistance to quinoxalines, quinolones, tigecycline, nitrofurantoin, and chloramphenicol (Li et al., 2019).

Multiple Mex efflux components were detected in *Pseudomonas aeruginosa*, including *mexA*–*mexF* and *mexY*, which assemble into clinically relevant RND pumps such as MexAB-OprM, MexCD-OprJ, MexEF-OprN, and MexXY-OprM (Pérez-Vázquez et al., 2023). MexAB-OprM is a principal contributor to resistance in *P. aeruginosa*, with associated components linked to resistance to ceftazidime, imipenem, and ciprofloxacin, whereas MexXY-OprM is strongly associated with aminoglycoside resistance (Habib et al., 2025); pump overexpression typically increases resistance, and MexCD-OprJ overexpression has additionally been linked to decreased virulence (Alcalde-Rico et al., 2018). Additional efflux-associated genes detected included *mexW* (MexW-OprM), *mexI*, and *mexQ* (Díaz-Torres et al., 2024), as well as *mexV*, *mexP*, and *mexH*, which form MexVW-OprM, MexPQ-OprE, and MexGHI-OpmD complexes, respectively (Akter et al., 2025); the identification of *yajC*, implicated in biofilm-related membrane functions and reported as a regulatory element influencing efflux-associated antibiotic and disinfectant resistance, further supports the presence of coordinated resistance and persistence traits in this isolate (Top et al., 2024; Akter et al., 2025).

Several regulators and modulators of RND efflux systems were identified, including *mexZ*, *mexT*, *mexS*, and *nfxB*, which coordinate the expression of key resistance-associated operons in *Pseudomonas aeruginosa* (Morita et al., 2015). The *mexEF-oprN* operon is indirectly regulated by *mexS* and directly activated by *mexT*, whereas *mexZ* functions as a local repressor of *mexXY-oprA* and *nfxB* is linked to the regulation of the MexCD-OprJ system (Morita et al., 2015). In addition, the MuxABC-OpmB efflux pump, encoded by *muxA*, *muxB*, *muxC*, and *opmB*, was detected; this system confers resistance to antibiotics such as novobiocin, aztreonam, erythromycin, and tetracycline, with overexpression leading to increased resistance levels (Mima et al., 2009; Castanheira et al., 2022).

Efflux systems belonging to the SMR and MFS families were also identified at the species level. In *Klebsiella* strains, *kpnF/kpnE* (KpnEF) and *kpnG/kpnH* (KpnGH) encode efflux pumps associated with resistance to colistin, macrolides, tetracyclines, aminoglycosides, and fluoroquinolones, and may also contribute to biofilm formation (Srinivasan and Rajamohan, 2013; Srinivasan et al., 2014). Additional efflux-related genes included *amvA* in *Acinetobacter baumannii*, linked to resistance against disinfectants such as chlorhexidine and benzalkonium chloride and reported to be more highly expressed in environmental strains (Rostami et al., 2021; Short et al., 2021); *mdtQ*, characterized as an outer membrane factor associated with MDR (Wright et al., 2025); and *pmpM*, encoding an H^+^ antiporter efflux system (He et al., 2004). The SMR and MFS transporters *emrE* and *abeS*, together with *emrB* as part of the EmrAB-TolC tripartite system, contribute to resistance to quaternary ammonium compounds and antibiotics via active efflux (Nasie et al., 2012; Lytvynenko et al., 2016; Pushpker et al., 2022; Puértolas-Balint et al., 2020). Beyond structural pumps, multiple transcriptional regulators were detected, including *marA*, *soxR*, and a mutated *acrR*, which collectively promote derepression and overexpression of AcrAB-TolC under chemical and oxidative stress (Alekshun and Levy, 2007; Poole, 2012; Harmon and Ruiz, 2022). Other regulators such as *CRP*, *H-NS*, *adeL*, *rsmA*, and *emrR* further modulate resistance-related pathways, often through integration with two-component systems (Nagakubo et al., 2002; Fernández et al., 2010; Yoon et al., 2013; Romero et al., 2016; Wang S. et al., 2021; Tian and Wang, 2023).

Efflux- and regulation-associated determinants were prominent across the isolates, particularly within Gram-negative systems that shape antibiotic penetration and intracellular drug accumulation. Several two-component systems (TCS) linked to environmental sensing and antimicrobial stress responses were detected, including BaeSR, ParRS, and CpxAR. In BaeSR, BaeS acts as the kinase sensor and BaeR as the response regulator; BaeS responds to external chemical and physical cues, such as increased concentrations of indole, copper, zinc, ethanol, sodium tungstate, tannins, and flavonoids and BaeR subsequently modulates transcription of downstream loci, including *acrD*, *mdtABC*, and *mdtD*, with implications for resistance phenotypes, including β-lactams and ampicillins, partly through effects on outer membrane proteins (Nagakubo et al., 2002; Wang S. et al., 2021; Liu et al., 2023). ParRS is similarly activated by perturbations in the surrounding environment under antimicrobial exposure (e.g., aminoglycosides and polymyxins), regulating determinants such as *oprD* and the MexXY-OprM efflux system (Fernández et al., 2010; Wang et al., 2013). CpxAR, characteristic of Gram-negative pathogens including *P. aeruginosa*, responds to diverse stresses and can also promote antibiotic resistance by activating the *mexAB-oprM* promoter (Tian and Wang, 2023).

Fluoroquinolone resistance-related genes included plasmid-associated *qnr* determinants (*qnrB19* and *qnrE1*), which protect DNA gyrase and topoisomerase IV, key enzymes in DNA replication and transcription from fluoroquinolone activity (Piddock, 1998; Redgrave et al., 2014; Albornoz et al., 2017). Notably, *qnrE1* has been described as a relatively recent fluoroquinolone resistance gene, and both *qnrB* and *qnrE* families are phylogenetically close and are suggested to be plasmid-transferred, with the *qnr* family largely reported in Enterobacteriaceae (Albornoz et al., 2017; Wang C. et al., 2021). In addition, *abaQ* was identified as an *Acinetobacter*-specific quinolone resistance determinant encoding a 434-aa MFS transporter with 12 transmembrane helices; *abaQ* functions as a DHA1-class H^+^ antiporter and appears selective for quinolones via efflux, without conferring broad multidrug resistance (Pérez-Varela et al., 2018; Attia and Elbaradei, 2019; Vivekanandan et al., 2025).

Beyond quinolones, several additional resistance mechanisms were represented across the dataset. For β-lactams, SHV-61 was identified among ESBL-related determinants, consistent with hydrolysis of a broad range of β-lactam antibiotics through covalent interaction with the β-lactam ring (Effendi et al., 2022; Rice, 2012; Liakopoulos et al., 2016), while OKP-A-12 and ADC-18 reflected chromosomally encoded β-lactamases characteristic of *Klebsiella* and *Acinetobacter*, respectively (Rice, 2012; Bhattacharya et al., 2014; Liakopoulos et al., 2016; Bellini et al., 2022); PDC-565 and *blaPAO* were aligned with *Pseudomonas* spp. (Jin et al., 2022; Le Terrier et al., 2025), and OXA-type β-lactamases (OXA-500, OXA-486, OXA-646) were included as class D enzymes that may be chromosomal or mobile-element borne (Tian et al., 2018; Mora-Ochomogo and Lohans, 2021), alongside ACT-156 with weaker carbapenem activity (Stewart et al., 2025). Bacitracin-associated determinants converged on lipid A biosynthesis/modification (*eptB*, *arnT*, *arnA*, *lpsB*), altering membrane charge and susceptibility (Needham and Trent, 2013; Tajer et al., 2024; van Duijkeren et al., 2018; Hood et al., 2013; Liu et al., 2025). Aminoglycoside resistance genes encoded modifying enzymes (AAC/ANT/APH pathways) and included *ant(3”)-IIa*, *aadA2*, *aph(3’)-IIb*, *aph(6)-Id*, and *aph*(3”)-Ib (Cameron et al., 2018; Zhang et al., 2023). Fosfomycin resistance-related findings included *fosA* (with *fosA6*/*fosA2* variants) and the uptake-associated transporter *uhpT* (Ito et al., 2017; Wang Y.-P. et al., 2022; Mattioni Marchetti et al., 2023; Cattoir et al., 2020; Herrera-Espejo et al., 2025). Finally, biocide/disinfectant-associated genes (*qacE*Δ*1*, *triB*, *triC*) were detected and have been linked to protection against quaternary ammonium compounds and resistance to triclosan/detergents, potentially co-occurring with other AMR determinants (Liu et al., 2024; Ntshonga et al., 2025; Tigabu et al., 2025). Other resistance genes—trimethoprim (*dfrA*), sulfonamides (*sul*), phenicols (*cat*), and elfamycin (EF-TU)—were present but limited in number and restricted to a subset of isolates, suggesting more discrete contributions compared with the larger resistance modules described above.

In this dataset, the trimethoprim resistance gene *dfrA12* was detected only in the KP isolate, mirroring the pattern observed for *sul1*, which confers sulfonamide resistance. Although chromosomal mutations can contribute to reduced antimicrobial susceptibility, these findings are more consistent with the acquisition of resistance determinants via mobile genetic elements as the dominant mechanism for both trimethoprim- and sulfonamide-associated genes (Sánchez-Osuna et al., 2020; Kneis et al., 2023). Mechanistically, *dfr* genes encode non-allelic variants of dihydrofolate reductase (DHFR), enabling folate metabolism to proceed despite trimethoprim inhibition, whereas *sul1* encodes an alternative dihydropteroate synthase (DHPS) that remains functional in the presence of sulfonamides; both pathways ultimately sustain folate (vitamin B9) biosynthesis, which is essential for DNA synthesis and broader cellular metabolism (Mazurek-Popczyk and Baldy-Chudzik, 2021; Huang et al., 2004; Jiang et al., 2019; D’Aimmo et al., 2023).

Notably, *dfrA12* and *sul1* were identified as part of a class 1 integron (*intI1*), specifically the cassette array *dfrA12–aadA2–qacE*Δ*1–sul1*, which is linked to multidrug resistance and has been associated with *IncF* plasmids; all of these elements were detected in KP in this study (Antunes et al., 2005; Chung et al., 2015; Domínguez et al., 2019). Additional resistance determinants included *catB7* in KP, which encodes a chloramphenicol acetyltransferase that inactivates chloramphenicol by acetylating the 3’-hydroxyl, thereby preventing inhibition of protein synthesis (Ghafoori et al., 2021). Beyond KP, an elfamycin-associated determinant (EF-TU) was identified, consistent with resistance arising through structural alterations in EF-TU that reduce elfamycin binding to functional domains (Yarlagadda et al., 2020; Marathe et al., 2023). Similarly, *tet*(39), detected in the analyzed AP isolate and regulated by *tetR*, is consistent with tetracycline resistance mediated by efflux and has been reported as prevalent among *Acinetobacter* strains associated with aquatic environments (Agersø and Guardabassi, 2005). Finally, CARD classifies genes and proteins into drug classes based on published phenotype associations, which explains why several determinants were categorized across aminoglycosides, fosfomycin, fluoroquinolones, aminocoumarins, macrolides, tetracyclines, β-lactams, and other classes in this analysis (Yao and Yiu, 2019).

### 4.2 Identified plasmids

The discovered plasmids are all characteristic of the *Enterobacteriaceae* family and are widely found in their genomes (McMillan et al., 2020). All of the identified plasmids are also a part of the same group of incompatible plasmids, which share mechanisms, replicons, or outdated terminology; most of them are denoted by the Inc suffix as their common identifier, with the exception of Colicinogenic plasmids (Col) (Rozwandowicz et al., 2018; Garcillán-Barcia et al., 2023).

*IncF* plasmids are a type of narrow-range plasmid, meaning they are only transferred and maintained by closely related species (Pitout and Chen, 2023). They carry a variety of ARGs that can be disseminated due to the many subtypes, mainly *IncFIB/FIA*, with *IncFIB(K)* detected in this study for both *Klebsiella* strains (Wang X. et al., 2021). *IncFIB* is known to facilitate conjugation mechanisms and the mobilization of resistance genes, including β-lactams, aminoglycosides, sulfonamides, and tetracyclines, as well as resistance to heavy metals such as mercury, tellurium, copper, silver, and arsenic (Stein et al., 2024).

*IncR* is another type of incompatibility plasmid, ranging from 40 to 160 kb, that is non-conjugative due to the complete absence of genes required for conjugation (Zhang et al., 2025). This is why, in many cases, this plasmid may fuse with other plasmids that possess said abilities, such as the *IncF* or *IncN* variants (Fang et al., 2024). *IncR* plasmids are efficiently transferable and are frequently associated with multidrug resistance, mainly conferring resistance to aminoglycosides (Elena et al., 2020; Zhang et al., 2025). It also possesses unique maintenance systems, stability-associated genes, and a high number of insertions, which may increase resistance and make it more difficult to elucidate (Zhang et al., 2025).

*IncY* is a newly characterized plasmid from the incompatibility group, whose natural host is *E. coli. This* is a type of prophage that replicates as an autonomous plasmid, measuring ∼90–100 kb; *IncY* is a low-copy-number plasmid and has been confirmed as the carrier of the *blaSHV-2* gene (Rozwandowicz et al., 2018; Zalewska et al., 2024). The *IncY* group is associated with β-lactams, aminoglycosides, fluoroquinolones, tetracycline derivatives, sulfonamides, and ampicillin resistance (Rozwandowicz et al., 2018; Zalewska et al., 2024). These types of incompatible plasmids are associated with *IncF, IncCI*, and/or *IncHI2*, and it has been suggested that they are likely to disseminate to other members of the *Enterobacteriaceae* family (Rozwandowicz et al., 2018; Kurittu et al., 2021; Zalewska et al., 2024). However, they have been detected only in *Salmonella* Typhi and *Escherichia coli*, and, compared with our analysis, the identified plasmids do not correlate with these hosts (Kurittu et al., 2021; Zalewska et al., 2024). Therefore, our findings suggest a possible dissemination of IncY plasmids into Klebsiella species, indicating potential interspecies transfer within environmental bacterial communities.

*Col* plasmids are categorized as incompatible gene plasmids, a type of coligenic plasmid. *ColE10*, specifically, is not transferred by conjugation and has not been widely described (Rozwandowicz et al., 2018; Zelendova et al., 2023; Garcias et al., 2025). However, *ColE10* plasmids have been suggested as an environmental contamination indicator, as they carry the *mcr-4* and mcr-5 genes, which confer plasmid-mediated colistin resistance (Rozwandowicz et al., 2018; Zelendova et al., 2023; Garcias et al., 2025). And *ColE* is directly associated with quinolone resistance and the spread of *qnrB* genes, such as *qnrB19* (Rozwandowicz et al., 2018; Juraschek et al., 2022).

### 4.3 The meaning of ARGs in the resistomes of pathogenic bacteria from the Yaque del Norte River

The Yaque del Norte River is one of the main rivers in the Dominican Republic, passing through several provinces and cities in the north, and is the source of many other affluent rivers that cover the rest of the national territory (Dirección de Información Ambiental y Recursos Naturales, 2016). This is why the constant contamination of pollutants poses a serious health risk, allowing the propagation of antibiotics, heavy metals, and other contaminants that bacteria have adapted to and proliferated due to resistance mechanisms (United Nations, 2025).

This is evidenced by the physical-chemical analysis results, which exceeded the established national and international thresholds. The BOD value for Point A is within the national range of 2–5 mg/L, but at an international level, this value, along with the values of Points B and C, still indicate moderate pollution in the river; and Points D and E are considered to be very contaminated in both cases (Ministerio de Medio Ambiente y Recursos Naturales, 2012; Li and Liu, 2019). The EC value for Point E is the only one considered above the limit at a national level (< 1,000 μS/cm), but internationally, the value for Point D is also above the limit of 800 μS/cm (Gobierno de la República Dominicana, 2021; Latif et al., 2024; World Health Organization [WHO], 2024a). Lastly, TDS values fall within the 1,000 mg/L limit set by the DR guidelines, but internationally, Point E exceeds the WHO maximum of 500 mg/L (Ministerio de Medio Ambiente y Recursos Naturales, 2012; Latif et al., 2024). These values, along with the previously mentioned pH, temperature, and ORP, the latter of which is below accepted ranges, indicate a limited-oxygen environment with high organic material, which supports the proliferation of facultative anaerobic microorganisms, especially enterobacteria (Pleto et al., 2020).

Although the environmental conditions observed in the river may favor microbial persistence, the isolated bacteria remained susceptible, with the exception of KP, to all the antibiotics used in the MIC assay; this does not mean that the risk of eventually developing resistance is not a possibility. Both *Klebsiella* species, EM and PA, still harbor extensive resistomes with numerous MDR genes, which could be expressed under appropriate stress conditions. Especially when several strains from this genus are known opportunistic pathogens, as stated by the WHO’s Bacterial Priority Pathogen List, that harbor most of the same AGRs (American Academy of Microbiology, 2009; Wang Y. et al., 2022; World Health Organization [WHO], 2024b). And the PathogenFinder results classify them as pathogens with a high likelihood of becoming pathogenic toward humans. As for the *Acinetobacter* species, although they possessed few ARGs, they were still classified as pathogens because the ratio of pathogenic to non-pathogenic genes/protein families matched that of other strains. Suggesting the presence of genomic features associated with pathogenic potential, but are still susceptible to some antibiotic treatments, which lowers their overall risk toward humans.

It is also of high importance to recognize the limitations of the genomic tools CARD and ResFinder, which classify “resistant genes” based on non-relevant clinical antibiotics and porins with low antibiotic resistance capacity, as exemplified by the not-defined *msbA* gene (Amábile-Cuevas and Lund-Zaina, 2024). Moreover, many of the classified genes could be genetically intrinsic or taxonomically/phylogenetically linked to the strain, and the *Enterobacteriaceae* family is no exception (American Academy of Microbiology, 2009; Wang Y. et al., 2022; Amábile-Cuevas and Lund-Zaina, 2024). Therefore, this and inactive genes (non-expressed) explain why a “resistant” strain has the proteins or genes to withstand antibiotic stress, but in practice, it does not show the capability to do so (American Academy of Microbiology, 2009; Amábile-Cuevas and Lund-Zaina, 2024). Despite this, ResFinder’s newest update shows the phenotypic resistance that the isolates might have, and in the case of the bacteria isolated in this study, the predicted phenotypic resistance and the results of the MIC assay agree with each other. Specifically, for the KP strain, which presented resistance in both ResFinder and the MIC assay to ciprofloxacin (*oqxAB* and *qnrB19*), trimethroprim (*oqxAB* and *dfrA12*), and sulfomethoxazole (*sul1*). While KQP presented predicted phenotypic resistance to one type of quinolone and one type of aminoglycoside.

It is also possible that this river environment may provide conditions that could facilitate horizontal gene transfer, as evidenced not only by the determination of conjugative and mobility plasmids, but also the identification of a novel plasmid in species yet to be reported. Allowing for the possibility that commensal bacteria can become pathogens and that pathogens can upgrade their resistomes and become multiresistant.

Some detected determinants may reflect intrinsic genomic features or annotation artifacts, as previously discussed for large resistance databases. This interpretation was based on quality-control metrics generated by the Staramr pipeline, which showed that some strains did not pass. Some resistance determinants may represent intrinsic genomic features or low-expression genes that do not necessarily translate into phenotypic resistance. These findings reinforce concerns regarding the presence of pathogenic microbes; since the river is utilized in agronomic irrigation systems and potable water that are in direct contact to other organisms such as plants, animals, and humans; arising the propagation of pathogenic entities, which could have potential implications for environmental and public health, and the industries related to them, including the agricultural industry (Jenkins et al., 2017; Laborda et al., 2022; Islam et al., 2024).

When comparing this data with previous reports and studies in the Dominican Republic, a pattern that may warrant further investigation. Data from the last 5 years released from PROMESE/CAL highlights antibiotics as highly demanded by the Dominican population in 2024, and reports from 2021 include amoxicillin (penicillin), amoxicillin-clavulanic acid combination (penicillin/β-lactamase inhibitor), and azithromycin (macrolide) as the most dispensed antibiotics (Programa de Medicamentos Esenciales/Central de Apoyo Logístico [PROMESE/CAL], 2021, 2024). In other main rivers in the country, Calderón et al. (2021) demonstrated the presence of resistant *Enterobacteriaceae* in the Isabela River, as well as Bonnelly et al. (2023), who found similar profiles in the Ozama River, both related to a high charge of urban pollutants, coupled with the rising mining industries, and other anthropogenic activities (Urgilez, 2024). The resemblance in resistance and antibiotic use patterns could therefore be attributed to the dispersion of these bacteria across the country’s major hydrographic basins.

The Dirección de Epidemiología (DIEPI) already issues weekly epidemiological alerts regarding the growing problem of pathogenic and antibiotic-resistant microorganisms, which are supported by our findings (Ministerio de Salud Pública, 2025). However, we still encourage the proper authorities to strengthen the established norms by integrating the necessary government entities and ensuring optimal collaboration that will lead to the best possible outcome for the Dominican population. This would consist of a i) more consistent monitoring of the quality and microbiological charge of not only the Yaque del Norte River, but also the other major affluents of the Dominican territory; ii) a well detailed and up-to-date report with the physical-chemical parameters of the rivers, along with its accepted ranges, that it is also reachable for the common folk and investigators; and finally iii) public recognition of the issue with its possible immediate and future impact on the surrounding areas.

It is important to acknowledge that this study analyzed a limited number of culturable isolates from the river. Therefore, the results should be interpreted as preliminary genomic evidence of the environmental resistome rather than a comprehensive ecological characterization of antimicrobial resistance across the entire river system. Future studies incorporating larger sample sizes and metagenomic approaches would provide a more detailed understanding of ARG diversity and dissemination in this ecosystem.

## 5 Conclusion

With the results it was confirmed that the Yaque del Norte River contains bacteria with broad resistomes, specifically *Pseudomonas aeruginosa, Klebsiella pneumoniae, Klebsiella quasipneumoniae, Acinetobacter pittii, Acinetobacter schindleri* HOEPLIER (a new strain) and *Enterobacter mori.* Revealing that each genome harbors multiple resistance-associated genes, as well as specific gene resistance toward other antibiotics, and an above 70% likelihood of becoming human pathogens.

As for the identified plasmids, they were only detected in the *Klebsiella* species, specially the *IncY* plasmid in the KP strain, a plasmid previously reported mainly in *Escherichia coli* and *Salmonella Typhi*, suggesting the dissemination of novel plasmids into other strains which in turn means a larger possibility for propagation of antibiotic resistance. Together with physicochemical parameters such as nutrient availability, oxygen levels, pH, and temperature, the river might be turning into a microbial reservoir with high pathogenic potential, which aligns with the recent warnings from the WHO about prioritary pathogens and their capacity to transfer genes to other organisms. Regardless, the identified bacteria, with the exception of KP, cannot be classified as resistant bacteria, since they were still susceptible to the antibiotic formulations they were exposed to.

This all highlights future public health risks that may arise due to pathogenic bacteria harboring multidrug resistance, since the Yaque del Norte River is essential for the region in terms of agriculture, energy, and potable water. These findings highlight the importance of strengthening environmental AMR monitoring strategies in the region, aiming to reduce the propagation of resistant microbial species. Nonetheless, this work represents the first genomic analysis conducted in the Yaque del Norte River, providing novel evidence of bacterial diversity and the genetic mechanisms involved in the resistome dynamics in the aquatic ecosystems in the Dominican Republic.

## Data Availability

The genomic sequencing data generated in this study are publicly available in the NCBI repository under BioProject accession PRJNA1395590, with associated BioSample and Sequence Read Archive (SRA) accessions.
